# Pandemic (H1N1) 2009 Encephalitis in Woman, Taiwan

**DOI:** 10.3201/eid1710.110916

**Published:** 2011-10

**Authors:** Aristine Cheng, Kuei-Hong Kuo, Chia-Jui Yang

**Affiliations:** Far Eastern Memorial Hospital, New Taipei City, Taiwan (A. Cheng, K.-H. Kuo, C.-J.Yang);; National Taiwan University Hospital and College of Medicine, Taipei, Taiwan (A. Cheng)

**Keywords:** viruses, influenza, encephalitis, pandemic (H1N1) 2009, Taiwan, dispatch

## Abstract

We report an unusual case of pandemic (H1N1) 2009–related encephalitis in an immunocompetent woman. Although rare cases of pandemic (H1N1) 2009 associated with encephalitis have been reported previously, in this patient, direct viral invasion of the central nervous system was shown by simultaneous detection of viral RNA and pleocytosis.

Neurologic complications of pandemic (H1N1) 2009 were first reported in children in May 2009 (*1*). Most subsequent reports were also of cases in children, because those <17 years of age appear most vulnerable (*2*). To our knowledge, only 1 retrospective review has documented the frequency of neurologic dysfunction among adults with pandemic (H1N1) 2009 (*3*). In this Asian cohort of 826 hospitalized adults, seizures developed in 6 of the 9 persons with neurologic manifestations, and none had encephalopathy or encephalitis. Thus, seizures appear to be the most common reason for seeking care for children and adults, followed by encephalopathy, in particular, acute necrotizing encephalopathy in children (*4*). We performed a Medline search of literature in English and found 5 case reports of adults with pandemic (H1N1) 2009 encephalitis. Here, we report a woman who sought care because of focal neurologic deficit.

## The Patient

The patient was a previously healthy 60-year-old housewife of Mandarin Chinese descent with well-controlled essential hypertension. Her regular medications consisted of once-daily doses of bisoprolol, irbesartan-chlorothiazide, and lercanidipine. She lived in a bungalow in New Taipei City, Taiwan, with her daughter and husband, who both remained asymptomatic. No travel, animal or insect contact, or influenza vaccination history was elicited.

Sore throat, epiphora, and otalgia developed 3 days before hospital admission to Far Eastern Memorial Hospital, New Taipei City. High sustained fever (up to 40°C) and abrupt left lower face paresthesias prompted a clinic visit the following day. Her family physician performed a rapid influenza diagnostic test, which had negative results. Nevertheless, she received empirical oseltamivir, according to the Taiwan Department of Health’s emergent initiatives for the peak pandemic (H1N1) 2009 season. After 3 doses of oseltamivir, she remained febrile, with progressive anorexia, malaise, and dizziness, and sought care at the emergency department of a medical center.

At the emergency department (day 4 of symptoms), acute urinary retention developed in the patient without anal sphincter involvement or saddle paresthesias. Despite sensing urgency of her full bladder, she was unable to void spontaneously, necessitating catheterization. She exhibited no other neurologic deficits, seizures, altered mentation, or meningism. Her respiratory symptoms remained mild and confined to the upper respiratory tract. Myalgia, arthralgia, and gastrointestinal symptoms were not prominent.

On admission, she had a temperature of 38.5°C but appeared well. Her blood pressure, pulse, and respiratory rate were 125/64 mm Hg, 89 beats per minute, and 20 breaths per minute, respectively. Results of her physical examination were unremarkable except for the relative bradycardia (which may have been attributable to β-blocker use) and an above average body mass index of 28 kg/m^2^. A neurologic examination found diminished sensation to light touch over the distribution of her left trigeminal mandibular nerve. Her other cranial and peripheral nerve functions and higher cortical functions were grossly intact. Ear, nose, and throat examination revealed the normal appearance of bilateral eardrums and pharynx.

Initial hemogram showed borderline leukocytosis (10,340 cells/μL) with relative lymphopenia (17% lymphocytes) and a platelet count within reference range (210 × 10^3^/μL). Biochemical testing revealed renal function, electrolytes, serum alkaline phosphatase, and transaminase levels within reference ranges. Plain chest radiographs and results of urinalysis were unremarkable. Electroencephalogram showed symmetric basal activities without epileptiform discharges. Magnetic resonance imaging (MRI) of the brain on day 9 of symptoms showed multifocal scattered T2 high-signal lesions over bilateral hemispheres, occipital horn, basal ganglion, and brainstem, involving both gray and white matter, with diffusion restriction (Figure, panel A). The ventriculitis correlated with her acute urinary retention.

**Figure:**
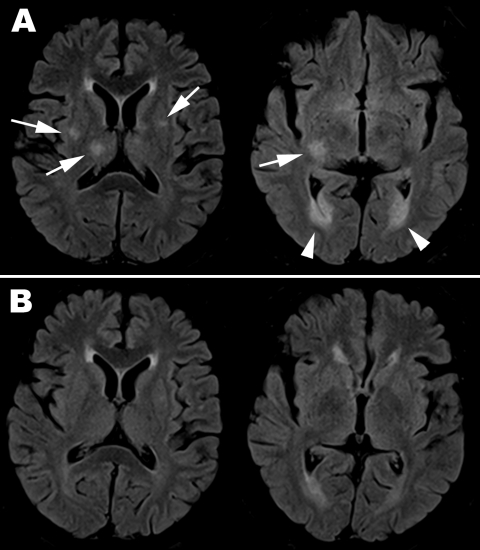
Magnetic resonance imaging with fluid-attenuated inversion recovery sequence of brain for adult patient with pandemic (H1N1) 2009 encephalitis, Taiwan. A) On day 9 after symptom onset, scattered asymmetric focal hyper signal intensities over bilateral putamen and right thalamus (arrow on the left image) and ventriculitis over bilateral occipital horns (arrow head over right image) are seen. B) By day 24, the lesions had resolved.

Cerebrospinal fluid (CSF) analysis was performed on days 8 and 11 (Table). Transition from a neutrophilic to a lymphocytic predominant picture was observed. CSF cryptococcal antigen test; Gram, India ink, and acid-fast stains; and subsequent bacterial, mycobacterial, and fungal cultures all yielded negative results. By real-time PCR screening of a wide panel of possible infectious etiologic agents, influenza A (nonsubtyped) was simultaneously identified in CSF, nasopharyngeal swab specimen, and blood on day 11.

A regimen of oseltamivir (75 mg 2×/d), initiated at onset of fever, was completed after 5 days. Defervescence occurred slowly over 2 weeks. Urodynamic study revealed detrusor spasticity, which was successfully treated with a combination of diazepam and phenazopyridine. Repeat brain MRI on day 24 showed near total resolution of the T2-hyperintense lesions (Figure, panel B). However, after 2 months, the patient reported residual left facial numbness.

## Conclusions

According to the Centers for Disease Control in Taiwan, the circulating influenza A strain for the winter-spring season of 2011 was pandemic (H1N1) 2009 with few exceptions (*5*). The pandemic signature of novel subtype H1N1 has been its predilection for infecting healthy adults and its high transmissibility; hence, we were unable to trace contact history in the case described here. The patient did not have known comorbid risk factors; hence, her clinical course was mild. Epidemiologic clues to her diagnosis included residence in an area with the highest incidence of pandemic (H1N1) 2009 in Taiwan in 2011 and her naive immunity (lacking vaccination or exposure to the pandemic 1918 strain). Clinical clues supporting pandemic (H1N1) 2009 infection include initial leukocytosis as opposed to leukopenia, relative lymphopenia, and initial false-negative rapid influenza diagnostic test (*6*).

Only a few cases of pandemic (H1N1) 2009 encephalitis in adults have ever been reported (Table A1). All patients had altered mental status with or without seizures. The onset of neurologic symptoms usually occurs within a few days of influenza-like illness. Unlike children, for whom the mortality rate can be as high as 30% (*4*), most adults survive, despite varying degrees of sequelae. Initial neurologic severity parallels the severity of pulmonary disease and is predictive of neurologic outcomes. This case highlights the possibility that subtle neurologic deficits may lead to underrecognition of the milder spectrum of central nervous system (CNS) complications associated with influenza. The patient’s report of focal paresthesias and micturition difficulties (in the absence of global neurocognitive defects) understates the substantial, albeit transient, CNS inflammation captured on serial CSF and MRI studies. MRI patterns of CNS inflammation in adults appear nonspecific, with T2 lesions distributed across both white and gray matter, with or without symmetry, brain necrosis, infarct, hemorrhage, edema, or ventriculitis.

This patient may be the eldest and only female adult reported with pandemic (H1N1) 2009 encephalitis. As observed, other adults were men from 20 to 40 years of age (Table A1). There may be a yet unidentified genetic predisposition for influenza-related encephalopathy to develop among Asians, as noted previously in children (*4*) (and possibly among male adults). A remarkable feature of our case was the simultaneous CNS detection of virus and pleocytosis, which suggests that the pathogenesis of subtype H1N1 encephalitis may not be simply due to immune activation or cytokine storm as current favored hypotheses propose but also may be caused by direct viral invasion. One possible mechanism is that the virus crosses the blood–brain barrier by way of the peripheral nerves (*12*). Although we cannot confirm the entry portal of the virus, we note that when the patient sought care, she had a peripheral cranial nerve deficit. Influenza A virus has been detected in the CSF of a small minority of Japanese children (especially in those with severe brain pathology) (*13*) and in 1 teenager in South Korea (*14*). In children, pleocytosis has rarely been described, whereas in this adult series, mild pleocytosis appears not infrequently (Table A1). Despite the 2008 recommendation of the Infectious Diseases Society of America regarding routine lumbar puncture in the management of encephalitis for survey of possible etiologic agents (*15*), CSF reverse transcription PCR for influenza is infrequently performed in adults (Table A1). Therefore, the role of viral CNS invasion may be underestimated.

In conclusion, physicians (not just pediatricians) should be alert to the possibility of neurologic disease due to pandemic (H1N1) 2009, especially in persons whose symptoms are subtle. Further studies are warranted to clarify and confirm the neurotropism, particularly for persons of Asian heritage, of pandemic (H1N1) 2009.

## Appendix

**Table A1:** Summary of published case reports for adults (age >18 y) in whom influenza subtype H1N1–related encephalopathy or encephalitis developed*

Authors, publication year (reference)	Age, y/sex	Ethnicity/nationality	RTI S/S	ILI to CNS, d	Neurologic S/S	Respiratory PCR	CSF PCR	CSF findings	MRI findings	Outcome
Akins et al., 2010 (*7*)	20/M	Hispanic	RF	5	Seizure, coma	Influenza A (H1N1)	No	Mild pleocytosis (leukocyte 53: erythrocyte 6)	Malignant brain edema, with T2-hyperintensities of white matter, symmetric	Mildly disabled (rigidity)
Wang et al., 2011 (*8*)	22/M	Asian	URI	3	Seizure, dysarthria, monoplegia	Influenza A (H1N1)	ND	No pleocytosis (leukocyte 0: erythrocyte 1)	ADEM-like lesion in white matter, symmetric	Full recovery
Ito et al., 2011 (*9*)	26/M	Asian	URI	1	Drowsiness, amnesia, confusion	Influenza A (H1N1)	ND	Mild pleocytosis (leukocyte 38: erythrocyte 3)	ADEM-like lesions in white matter, symmetric	Full recovery
Chen et al., 2010 (*10*)	40/M	Asian	RF	2	Seizure, hemiplegia	Influenza A (H1N1)	ND	Mild pleocytosis (leukocyte 13: erythrocyte NA)	Regional gray matter T2-hyperintensities, asymmetric	Moderately disabled (hemiplegia)
Fugate et al., 2010 (*11*)	40/M	American	RF	Not clear	Coma, left gaze deviation	Influenza A, not subtyped	ND	Hemorrhagic (leukocyte 1: erythrocyte 157)	Acute hemorrhagic encephalitis, symmetric	Severely disabled (vegetative)
This study	60/F	Asian	URI	2	Focal paresthesiae, urinary retention	Influenza A, not subtyped	Yes	Marked pleocytosis (leukocyte 244: erythrocyte 12)	Scattered white and gray matter T2-hyperintensites, asymmetric	Mild residual deficits

*RTI S/S, respiratory tract infection symptoms and signs; ILI to CNS, interval from influenza-like illness to central nervous system symptoms and signs; respiratory PCR, respiratory tract specimen (e.g., nasopharyngeal swab or broncheoalveolar lavage specimen) real-time PCR for influenza A virus; CSF PCR, cerebrospinal fluid specimen real-time PCR for influenza A virus; MRI, magnetic resonance imaging; RF, respiratory failure; leukocyte:erythrocyte, leukocyte to erythrocyte ratio/mm^3^; URI, upper respiratory tract infection; ND, not done; ADEM, acute demyelinating encephalomyelitis.

**Table:** Sequential cerebrospinal fluid analysis for adult patient with pandemic (H1N1) 2009 encephalitis, Taiwan*

Cerebrospinal fluid	Day 8	Day 11	Reference range
Protein	94	78	15–40 mg/dL
Glucose	61	61	40–70 mg/dL
Lactate	2.9	2.7	1–2 mmol/L
Leukocytes (Lymph:PMN)	244 (1:99)	64 (98:2)	0–5 L/mm^3^
Erythrocytes	12	22	0/mm^3^

*Lymph:PMN, lymphocytes:polymorphonuclear cells.

## References

[1] Centers for Disease Control and Prevention. Neurologic complications associated with novel influenza A (H1N1) virus infection in children—Dallas, Texas, May 2009. MMWR Morb Mortal Wkly Rep. 2009;58:773–8. 19629027

[2] Yildizdaş D, Kendirli T, Arslanköylü AE, Horoz OO, Incecik F, Ince E, Neurological complications of pandemic influenza (H1N1) in children. Eur J Pediatr. 2011;170:779–88. 10.1007/s00431-010-1352-y 21110204

[3] Tan K, Prerna A, Leo YS. Surveillance of H1N1-related neurological complications. Lancet Neurol. 2010;9:142–3. 10.1016/S1474-4422(10)70015-6 20129164

[4] Martin A, Reade EP. Acute necrotizing encephalopathy progressing to brain death in a pediatric patient with novel influenza A (H1N1) infection. Clin Infect Dis. 2010;50:e50–2. 10.1086/651501 20218891

[5] Centers for Disease Control, Department of Health. Notifiable infectious diseases statistics system. Taiwan, ROC [in Chinese] [cited 2011 May 20]. http://nidss.cdc.gov.tw/index.aspx

[6] Cunha BA, Pherez FM, Schoch P. Diagnostic importance of relative lymphopenia as a marker of swine influenza (H1N1) in adults. Clin Infect Dis. 2009;49:1454–6. 10.1086/644496 19824851

[7] Akins PT, Belko J, Uyeki TM, Axelrod Y, Lee KK, Silverthorn J. H1N1 encephalitis with malignant edema and review of neurologic complications from influenza. Neurocrit Care. 2010;13:396–406. 10.1007/s12028-010-9436-0 20811962 PMC7100075

[8] Wang J, Duan S, Zhao J, Zhang L. Acute disseminated encephalomyelitis associated with influenza A H1N1 infection. Neurol Sci. 2011 Mar 8; [Epub ahead of print]. 10.1007/s10072-011-0500-0 21384278

[9] Ito S, Shima S, Ueda A, Kawamura N, Asakura K, Mutoh T. Transient splenial lesion of the corpus callosum in H1N1 influenza virus-associated encephalitis/encephalopathy. Intern Med. 2011;50:915–8. 10.2169/internalmedicine.50.4147 21498942

[10] Chen YC, Lo CP, Chang TP. Novel influenza A (H1N1)–associated encephalopathy/encephalitis with severe neurological sequelae and unique image features—a case report. J Neurol Sci. 2010;298:110–3. 10.1016/j.jns.2010.09.010 20870249

[11] Fugate JE, Lam EM, Rabinstein AA, Wijdicks EF. Acute hemorrhagic leukoencephalitis and hypoxic brain injury associated with H1N1 influenza. Arch Neurol. 2010;67:756–8. 10.1001/archneurol.2010.122 20558397

[12] Wang GF, Li W, Li K. Acute encephalopathy and encephalitis caused by influenza virus infection. Curr Opin Neurol. 2010;23:305–11. 10.1097/WCO.0b013e328338f6c9 20455276

[13] Morishima T, Togashi T, Yokota S, Okuno Y, Miyazaki C, Tashiro M, Encephalitis and encephalopathy associated with an influenza epidemic in Japan. Clin Infect Dis. 2002;35:512–7. 10.1086/341407 12173123

[14] Moon SM, Kim SH, Jeong MH, Lee EH, Ko TS. Acute encephalopathy and pandemic (H1N1) 2009. Emerg Infect Dis. 2010;16:1811–3. 21029558 10.3201/eid1611.100682 PMC3294526

[15] Tunkel AR, Glaser CA, Bloch KC, Sejvar JJ, Marra CM, Roos KL, The management of encephalitis: clinical practice guidelines by the Infectious Diseases Society of America. Clin Infect Dis. 2008;47:303–27. 10.1086/589747 18582201

